# Improved inherited peripheral neuropathy genetic diagnosis by whole-exome sequencing

**DOI:** 10.1002/mgg3.126

**Published:** 2015-01-14

**Authors:** Alexander P Drew, Danqing Zhu, Aditi Kidambi, Carolyn Ly, Shelisa Tey, Megan H Brewer, Azlina Ahmad-Annuar, Garth A Nicholson, Marina L Kennerson

**Affiliations:** 1 Northcott Neuroscience Laboratory, ANZAC Research InstituteSydney, Australia; 2 Molecular Medicine Laboratory, Concord HospitalSydney, Australia; 3 Sydney Medical School, University of SydneySydney, Australia; 4 Department of Biomedical Science, Faculty of Medicine, University of Malaya50603, Kuala Lumpur, Malaysia

**Keywords:** Charcot-Marie-Tooth disease, genetic diagnosis, inherited peripheral neuropathy, whole-exome sequencing

## Abstract

Inherited peripheral neuropathies (IPNs) are a group of related diseases primarily affecting the peripheral motor and sensory neurons. They include the hereditary sensory neuropathies (HSN), hereditary motor neuropathies (HMN), and Charcot-Marie-Tooth disease (CMT). Using whole-exome sequencing (WES) to achieve a genetic diagnosis is particularly suited to IPNs, where over 80 genes are involved with weak genotype–phenotype correlations beyond the most common genes. We performed WES for 110 index patients with IPN where the genetic cause was undetermined after previous screening for mutations in common genes selected by phenotype and mode of inheritance. We identified 41 missense sequence variants in the known IPN genes in our cohort of 110 index patients. Nine variants (8%), identified in the genes *MFN2*, *GJB1*, *BSCL2,* and *SETX*, are previously reported mutations and considered to be pathogenic in these families. Twelve novel variants (11%) in the genes *NEFL*, *TRPV4*, *KIF1B*, *BICD2*, and *SETX* are implicated in the disease but require further evidence of pathogenicity. The remaining 20 variants were confirmed as polymorphisms (not causing the disease) and are detailed here to help interpret sequence variants identified in other family studies. Validation using segregation, normal controls, and bioinformatics tools was valuable as supporting evidence for sequence variants implicated in disease. In addition, we identified one *SETX* sequence variant (c.7640T>C), previously reported as a putative mutation, which we have confirmed as a nonpathogenic rare polymorphism. This study highlights the advantage of using WES for genetic diagnosis in highly heterogeneous diseases such as IPNs and has been particularly powerful in this cohort where genetic diagnosis could not be achieved due to phenotype and mode of inheritance not being previously obvious. However, first tier testing for common genes in clinically well-defined cases remains important and will account for most positive results.

## Introduction

Inherited peripheral neuropathies (IPNs) are a group of related diseases primarily affecting the peripheral motor and sensory neurons. They include the hereditary sensory neuropathies (HSN), hereditary motor neuropathies (HMN), and Charcot-Marie-Tooth disease (CMT). There is significant genetic and clinical heterogeneity in IPNs. To date, mutations have been described in over 80 genes that cause CMT and related disorders (Rossor et al. 2013; Saporta and Shy 2013; Siskind et al. 2013; Timmerman et al. 2014). Clinical heterogeneity includes both phenotypic variation between different families with mutations in the same gene (interfamilial) (Houlden et al. 2008) and variation within single families where all individuals carry the same gene mutation (intrafamilial) (Irobi et al. 2004). Mutations in different genes can also cause similar phenotypes (locus heterogeneity).

Locus heterogeneity of IPNs makes screening for mutations in known IPN genes time consuming and costly. Whole-exome sequencing (WES) has been shown to be an efficient tool for mutation screening CMT disease (Choi et al. 2012). The recent decrease in costs of WES has made it feasible to examine all known IPN genes in families. Furthermore, WES provides data that may be useful in identifying novel IPN disease genes in these families and also allows future querying of new disease genes as they are discovered. We have carried out a mutation screen of the known IPN disease genes using WES in 110 unrelated index patients with inherited peripheral neuropathy.

## Materials and Methods

### Standard protocol approvals, registrations, and patient consents

Individuals participating in this study were enrolled through the Neurogenetics Clinic Concord Hospital, Sydney. Genomic DNA was isolated from peripheral blood. These procedures were performed with informed consent according to protocols approved by the Sydney Local Health District, Human Ethics Committee, Concord Hospital, Australia (HREC/11/CRGH/105).

### Study participants

The 110 index patients were from families with multiple affected individuals except for two sporadic cases which could represent recessive inheritance or sporadic cases. Patient selection was from families with unknown genetic etiology after genetic testing for common IPN genes as described below. Index patients had various IPN phenotypes; CMT (*n* = 76), HMN (*n* = 14), HMN with pyramidal tract signs (HMNP; *n* = 15), and HSN (*n* = 1). Families with CMT were further classified into axonal CMT (CMT2; *n* = 43), demyelinating CMT (CMT1; *n* = 4), CMT with pyramidal signs (*n* = 2), and CMTX (*n* = 27). Families with no male-to-male transmission and with milder phenotype in females were classified as X linked (CMTX). However, due to the small size of these pedigrees, autosomal dominant inheritance with reduced disease penetrance could not be excluded.

### Genetic analysis

Prior to this study, patients were screened for mutations in common IPN genes based on clinical presentation. These genes included; MLPA duplication analysis of *PMP22* (MIM 601097) and Sanger sequencing for point mutations in *PMP22, MPZ* (MIM 159440)*, GJB1* (MIM 304040), *MFN2* (MIM 608507), *SPTLC1* (MIM 605712) and *SPTLC2* (MIM 605713). WES of DNA from index patients was outsourced to Axeq Technologies (South Korea). In brief, sequencing libraries were prepared using the Illumina TrueSeq kit and sequenced as 100 bp paired-end reads on the HiSeq 2000 Sequencer (Illumina, San Diego, CA). Sequence reads were aligned to the Human Genome Feb 2009 (GRCh37/hg19) assembly using BWA software (Li and Durbin 2009). Duplicate reads were removed using Picard software (http://picard.sourceforge.net/). Sequence variants (single nucleotide variants and indels) were called using SAMTOOLS (Li et al. 2009) and annotated using ANNOVAR (Wang et al. 2010). Annotated WES data were examined for variants in 75 IPN genes selected for relevance for the phenotypes in our cohort: *AARS* (MIM 601065), *AIFM1* (MIM 300169), *ARHGEF10* (MIM 608136), *ATL1* (MIM 606439), *ATP7A* (MIM 300011), *BSCL2* (MIM 606158), *CCT5* (MIM 610150)*, COX6A1* (MIM 602072), *DARS* (MIM 603084), *DCTN1* (MIM 601143), *DHTKD1* (MIM 614984), *DNAJB2* (MIM 604139), *DNM2* (MIM 602378), *DNMT1* (MIM 126375), *DYNC1H1* (MIM 600112), *EGR2* (MIM 129010), *FAM134B* (MIM 613114), *FBLN5* (MIM 604580), *FGD4* (MIM 611104), *FIG 4* (MIM 609390), *GARS* (MIM 600287), *GDAP1* (MIM 606598), *GJB1*, *GNB4* (MIM 610863), *HINT1* (MIM 601314), *HK1* (MIM 142600), *HSPB1* (MIM 602195), *HSPB3* (MIM 604624), *HSPB8* (MIM 608014), *IFRD1* (MIM 603502), *IGHMBP2* (MIM 600502), *IKBKAP* (MIM 603722), *INF2* (MIM 610982), *KARS* (MIM 601421), *KIF1A* (MIM 601255), *KIF1B* (MIM 605995), *LITAF* (MIM 603795), *LMNA* (MIM 150330), *LRSAM1* (MIM 610933), *MED25* (MIM 610197), *MFN2*, *MPZ*, *MTMR2* (MIM 603557), *NDRG1* (MIM 605262), *NEFL* (MIM 162280), *NGFB* (MIM 162030), *NTRK1* (MIM 191315), *PDK3* (MIM 300906), *PLEKHG5* (MIM 611101), *PMP22*, *PRPS1* (MIM 311850), *PRX* (MIM 605725), *RAB7A* (MIM 602298), *REEP1* (MIM 609139), *SBF1* (*MTMR5*) (MIM 603560), *SBF2* (*MTMR13*) (MIM 607697), *SETX* (MIM 608465), *SH3TC2* (MIM 608206), *SLC12A6* (MIM 604878), *SLC5A7* (MIM 608761), *SOX10* (MIM 602229), *SPTLC1*, *SPTLC2*, *SURF1* (MIM 185620), *TDP1* (MIM 607198), *TFG* (MIM 602498), *TRIM2* (MIM 614141), *TRPV4* (MIM 605427), *VAPB* (MIM 605704), *VCP* (MIM 601023), *WNK1* (MIM 605232), and *YARS* (MIM 603623). The sequence variants that were considered for further study included; nonsynonymous, frameshift, splicing, small indels, upstream promoter, and regulatory and UTR variants. The annotation and nomenclature of sequence variants were checked using the web based software Mutalyzer 2.0.beta-29 (Wildeman et al. 2008). Variants identified with WES were confirmed by Sanger sequencing PCR amplicons, using the sequencing service at the ACRF Facility (Garvan Institute, Australia). Where possible, variants were assessed for segregation with the disease in the respective families. Novel variants that segregated with the disease were then genotyped in 1000 chromosomes from a neurologically normal control panel using HRM genotyping assays and a 96-well LightScanner instrument (BioFire Diagnostics, Inc., Salt Lake City, Utah, USA.) as described previously (Kennerson et al. 2007). Sequence variants identified in these patients were submitted to the NCBI ClinVar database (http://www.ncbi.nlm.nih.gov/clinvar/).

### Bioinformatics analysis of variants

Novel variants were assessed to predict if they were likely to cause disease. DNA and protein sequences were obtained from the UCSC Genome Browser (GRCh37/hg19) (Kent et al. 2002). Conservation of nucleotides was assessed using GERP scores (Cooper et al. 2010) and Vertebrate conservation (100 vertebrates Basewise Conservation by PhyloP) (Pollard et al. 2010) which were accessed through the UCSC Genome Browser (Karolchik et al. 2014). The effect of amino acid-substitutions was assessed using the software SIFT (Kumar et al. 2009) and Polyphen2 (Adzhubei et al. 2010). Sequencing coverage of target genes was assessed using python scripts implementing the pysam wrapper for the SAMtools C-API (Li et al. 2009) and analysis in *R* (R Core Team 2010).

## Results

### Assessment of whole-exome sequencing in patients with peripheral neuropathy

WES was performed on 110 index patients from unrelated peripheral neuropathy families. Two samples with previously identified point mutations in *PMP22* and *MPZ* were included as positive controls in addition to the 110 unknown samples. The read depth of the aligned sequencing data is a measure of quality and sensitivity to detect mutations. All but one of the 110 samples achieved a mean read depth of >30X for the target regions (average 45.5X, range 27.5X–88X). Therefore, our WES data have >90% sensitivity to detect heterozygous variants within the exome target regions (Choi et al. 2009). The sequence coverage in our study is comparable to previous reports detecting heterozygous mutations in known IPN genes using WES (Hedges et al. 2009; Montenegro et al. 2011). The coverage of the target genes for the 112 samples is shown in supplementary Figure S1 panel A–D. Furthermore, blind analysis of the exome data for the two positive controls correctly identified the respective mutations, *PMP22* c.392C>G and *MPZ* c.371C>T (read depth 41.6X and 46.7X respectively).

### Identification of variants in known IPN genes

WES initially identified 62 sequence variants in 20 known IPN genes. Twenty variants were excluded as false positives by Sanger sequencing. Of the remaining 41 sequence variants, 21 were excluded as rare or novel polymorphisms by segregation analysis or from previously published evidence (Table S1). The proportion of sequence variants in IPN genes implicated in families is shown in Table1.

**Table 1:** Number of cases and proportion of sequence variants in IPN genes implicated according to inherited peripheral neuropathy phenotype

Disease classification	Families (*n*)	Previously reported variants	Novel sequence variants	Total variants
HMN	14	0	2 (1.8%)	2 (1.8%)
HMNP	15	2 (1.8%)	2 (1.8%)	4 (3.6%)
HMNX	2	0	0	0
HSN	1	0	0	0
CMT sporadic or unknown inheritance	2	0	0	0
CMT2	43	3 (2.7%)	7 (6.4%)	9 (8.2%)
CMT1	4	0	1 (0.9%)	1 (0.9%)
CMT with pyramidal signs	2	0	0	0
CMTX	27	4 (3.6%)	0	4 (3.6%)
TOTAL	110	9 (8.1%)	12 (11%)	21 (18.2%)

In 21 of the 110 index patients, we identified a sequence variant that caused a missense mutation in a known CMT or HMN disease gene (Table1). All sequence variants identified in genes causing HSN were confirmed as polymorphisms in our cohort. Nine sequence variants were previously reported (Table2) and a further 12 are novel putative pathogenic variants, not previously described in variant databases (dbSNP 137 and NHLBI ESP Exome Variant Server) or the literature (Table3). The previously reported sequence variants segregated with the disease in each respective family and were therefore considered to be the pathogenic gene mutation in these families. For the novel sequence variants, segregation with the disease phenotype in each family was assessed where possible. Novel sequence variants that segregated were then screened in 1000 neurologically normal control chromosomes. It should be noted that all families were not large enough to statistically show linkage to the respective IPN disease locus and functional analysis of the variants was beyond the scope of this study. We therefore cannot conclusively assign pathogenicity to the novel sequence variants. Bioinformatics analyses, assessing the conservation and predicting protein function disruption due to sequence variant changes, were used to further assess the likelihood of pathogenicity. Variants that were highly conserved and predicted to disrupt protein function were considered to be more likely pathogenic. The missense variants in known IPN genes and clinical information on the families carrying the variants are described in detail below.

**Table 2:** Previously reported pathogenic sequence variants identified in known peripheral neuropathy genes

Family ID	Disease classification	Gene	cDNA change	Amino acid change	dbSNP accession	Reference	Segregates with disease
A	CMT2	*MFN2*	c.775C>T	p.R259C	–	Angiari et al. (2006)	Yes
B	CMT2	*MFN2*	c.1090C>T	p.R364W	–	Chung et al. (2006), Zuchner et al. (2006)	Yes
C	CMTX	*GJB1*	c.77C>T	p.S26L	–	Yoshimura et al. (1996)	Yes
D	CMTX	*GJB1*	c.259C>G	p.P87A	–	Nelis et al. (1997)	Yes
E	CMTX	*GJB1*	c.580A>G	p.M194V	–	Silander et al. (1997)	Yes
F	CMTX	*GJB1*	c.790C>T	p.R264C	–	Numakura et al. (2002)	Yes
G	CMT2	*BSCL2*	c.263A>G	p.N88S	–	Windpassinger et al. (2004)	Yes
H	HMNP	*BSCL2*	c.263A>G	p.N88S	–	Windpassinger et al. (2004)	Yes
I	HMNP	*SETX*	c.8C>T	p.T3I	rs28941475	Chen et al. (2004)	Yes

The following GenBank accession numbers describe the genes and annotations in table: *MFN2*; NM_001127660.1, *GJB1*; NM_000166.5, *BSCL2*; NM_032667.5, *SETX*; NM_015046.5.

**Table 3:** Novel sequence variants in IPN genes inplicated in disease

Family ID	Disease Classification	Gene	cDNA change	Amino acid change	Vertebrate conservation	GERP	SIFT	Polyphen-2	Likely to cause disease
J	HMNP	*SETX*	c.1504C>T	p.R502W	2.15	3.86	nt	0.999	+
K	CMT2	*SETX*	c.4273A>G	p.K1425E	0.04	1.35	pt	0.001	−
L	CMT2	*SETX*	c.4225A>T	p.N1409Y	0.61	0.0559	nt	0.124	−
M	CMT2	*NEFL*	c.803T>G	p.L268R	4.33	5.62	nt	0.998	+
N	CMT2	*NEFL*	c.794A>G	p.Y265C	3.35	5.62	pt	1.00	+
O	CMT2	*NEFL*	c.1007T>C	p.L336P	5.90	5.1	nt	0.956	+
P	CMT2	*NEFL*	c.1319C>T	p.P440L	6.55	5.04	nt	0.551	+
Q	HMN	*TRPV4*	c.549G>C	p.E183D	0.74	3.69	pt	0.068	−
R	HMNP	*KIF1B*	c.2274G>T	p.E758D	1.53	4.67	nt	0.998	+
S	CMT2	*KIF1B*	c.4073T>C	p.V1358A	6.22	−2.14	nt	0.034	+/−
T	CMT1	*BICD2*	c.1079C>T	p.A360V	2.95	−0.249	pt	0.001	−
U	HMN	*BICD2*	c.1993G>T	p.V665L	3.10	2.52	pt	0.056	−

The following GenBank accession numbers describe the genes and annotations in this table: *NEFL*; NM_006158.4, *KIF1B*; NM_183416.3 for sequence variant c.2274G>T and NM_015074.3 for sequence variant c.4073T>C, *TRPV4*; NM_021625.4, *BICD2*:NM_001003800.1 and *SETX*; NM_015046.5. SIFT scores: nt, not tolerated; pt, predict tolerated. Likely to Cause Disease: +, supportive evidence implicated to cause disease; −, mostly unsupportive unlikely to cause disease; +/−, both supportive and negative results. Polyphen-2 scores approaching 1 are more confidently predicted to be deleterious. For the vertebrate conservation by PhyloP scores, positive values are predicted to be conserved and negative values are predicted to be fast-evolving. GERP scores >2 are considered to be evolutionarily constrained.

### Mutations in MFN2

Two previously reported *MFN2* mutations, c.775C>T (p.R259C) and c.1090C>T (p.R364W) (Chung et al. 2006; Zuchner et al. 2006), were identified in two index patients from Family A and B respectively (Table2). The p.R259C mutation segregated with the disease in a family with apparent autosomal dominant inheritance, adolescent onset, axonal motor neuropathy, and mild distal sensory loss. Two affected males developed clinical symptoms earlier than their female siblings. The p.R259C mutation has been previously reported in a three-generation pedigree with mild to moderate CMT2. The p.R364W mutation was identified in a family with an early onset, slowly progressing, demyelinating CMT. Previously this mutation was described causing hereditary motor and sensory neuropathy with optic atrophy (HMSN6; OMIM#601152) in two separate families (Chung et al. 2006; Zuchner et al. 2006). No optic atrophy was observed in our family suggesting the possibility of unidentified modifiers affecting the variability of clinical symptoms in this family. Both patients with *MFN2* mutations were last assessed prior to MFN2 testing being available in our laboratory (years 2007 and 1994 respectively) and no retrospective testing was requested in these cases.

### Mutations in GJB1

In this cohort, four previously reported missense mutations were identified for *GJB1;* c.77C>T (p.S26L) (Ohnishi et al. 1995; Yoshimura et al. 1996; Bone et al. 1997; Nelis et al. 1997; Oh et al. 1997; Haites et al. 1998; Hahn et al. 1999), c.259C>G (p.P87A) (Nelis et al. 1997), c.580A>G (p.M194V) (Silander et al. 1997) and c.790C>T (p.R264C) (Numakura et al. 2002) (Families C–F, Table2). The disease in three of the four families was not obviously X linked (Families C, E and F). While male-to-male inheritance was absent in all four families, affected males did not produce male offspring in three of the families. This created uncertainty in assessing the true likelihood of male-to-male transmission of the disease. In all families the males were more severely affected than the females, however, pedigree size was small.

Family C, with the p.S26L mutation, had two affected males presenting with a severe demyelinating CMT phenotype, and two older females with only a mild sensory neuropathy affecting the feet. The p.M194V mutation was identified in a small pedigree (Family E) in which a carrier female with mild peripheral neuropathy had two sons with a CMT1 phenotype. Family F, with the p.R264C mutation had only two affected females (mother and daughter), with no at-risk males. Both women had a juvenile onset axonal HMN phenotype presenting as weakness of the feet and ankles, with later progression to include pes cavus, hammertoes, and muscle wasting of the feet. There was no sensory involvement and normal nerve conduction velocities. As there were no affected males in this pedigree, it is unknown if males would present with a more severe phenotype than the females in the family. This mutation was previously reported to cause an axonal CMT phenotype (Numakura et al. 2002). In these families (C, E, and F), while you could discern phenotypic differences between male and female individuals, the pedigree information was insufficient at the time to suggest X-linked inheritance.

In Family D, with the p.P87A mutation, three affected males had a demyelinating CMT phenotype with slowed nerve conduction velocities. Their mother had a mild phenotype with distal weakness and pes cavus only. Due to the clear indications of CMTX in this family, the proband was previously diagnostically tested for *GJB1* mutations in 1997 but found to be negative. This testing was done using constant denaturant gel electrophoresis (CDGE) a method that can lead to false negatives if the mutations are located between melt domains or if signals are quenched by secondary SNPs (Hovig et al. 1991). The missed diagnosis is likely due to the reduced sensitivity of mutation screening used at the time.

### Mutations in BSCL2

The previously reported c.263A>G (p.N88S) mutation in *BSCL2 (Windpassinger* et al. 2004*)* was identified in index individuals from two unrelated families (Family G and H, Table2). The only two *BSCL2* mutations reported, p.N88S and p.S90L represent a mutational hotspot (Dierick et al. 2008) previously described in patients with HMN, Silver Syndrome, and CMT (Irobi et al. 2004; Windpassinger et al. 2004; Auer-Grumbach et al. 2005; Dierick et al. 2008). These two families presented with phenotypes similar to those previously reported. Patients from Family G had an early-onset HMNP, with a mild sensory neuropathy in some individuals and members were diagnosed as having HMN or CMT depending on their variable sensory symptoms. Patients from Family H had early-onset HMNP with no sensory involvement.

### Mutations in SETX

One previously reported mutation (Table2) and three novel mutations in *SETX* (Table3) were identified in four families. Mutations in *SETX* have been reported for ALS4 (Chen et al. 2004) which is a juvenile onset HMN with some clinical features similar to CMT2 (de Jonghe et al. 2002). In this study, we identified the previously reported c.8C>T (p.T3I) *SETX* mutation in a large multi-generational family with HMNP (Family I, Table2). The clinical features of Family I are similar to the family described by Chen, et al. with the same mutation. Family I has previously been reported as having peroneal muscular atrophy with pyramidal features and minor sensory involvement (Harding and Thomas 1984). The p.T3I variant segregates with four affected individuals and one at-risk individual, who was 3 years of age when clinically examined. The earliest known age of onset for disease symptoms in this family is 5 years and it is likely that this individual was too young to exhibit symptoms at the time of examination. Any further clinical assessment in this family is unknown. The family has similar ethnicity (German/Austrian) as the original family reporting the p.T3I mutation (de Jonghe et al. 2002; Chen et al. 2004). It is unknown whether this mutation in these two families is identical-by-decent or a recurring mutation hot spot.

**Figure 1:**
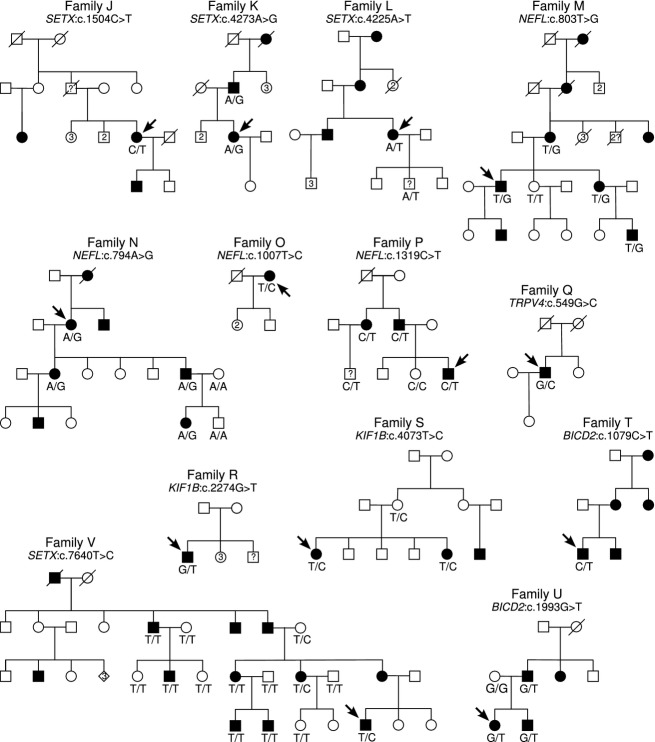
Segregation of novel sequence variants in families with CMT disease. Solid symbols indicate individuals with IPN. Genotypes are indicated below tested individuals. Arrows indicate the index individual initially sequenced using WES.

Three additional novel *SETX* sequence variants were also identified in our cohort. One variant, c.1504C>T (p.R502W) was identified in an index individual diagnosed with HMNP (Family J, Table3 and Fig.1). Only the proband was available for clinical examination and DNA analysis. She has an affected son and based on the patient's information of family history. Her father and cousin are also affected suggesting autosomal dominant inheritance. Conservation analysis and variant prediction programs suggested this variant is likely to be pathogenic (Table3). Family K (Table3 and Fig.1), a family with autosomal dominant axonal CMT, was found to carry c.4273A>G (p.K1425E) variant. The female proband presented with pes cavus, hammer toes, wasting of the muscles in the feet and hands, weakness in the muscles of the lower limbs and distal sensory loss. The variant was confirmed in the patient's father who presents with a similar affected phenotype. However, bioinformatics analysis of this variant indicated the nucleotide is not conserved, the glutamic acid residue is present at position 1425 in other species, and the p.K1425E amino acid substitution is predicted to be benign (Table3). Lastly, the sequence variant c.4225A>T (p.N1409Y) was identified in a CMT2 family with autosomal dominant inheritance (Family L, Table3 and Fig.1). The index patient presented with an axonal CMT, with disease progression involving slowing of motor and sensory nerve conduction velocities. In addition, the patient had a loss of balance and restless legs. DNA samples from the proband and her possibly affected son were analyzed, both of whom carried the c.4225A>T sequence variant. However, bioinformatics analysis suggested this variant is nonpathogenic (Table3). Previous mutations in *SETX* have only been reported in HMN and ALS4 (Chen et al. 2004). The bioinformatics analysis of the *SETX* variants in the two CMT families suggests they are unlikely to be pathogenic. However, additional evidence from family segregation is necessary to be conclusive.

### Mutations in NEFL

Novel *NEFL* sequence variants were identified in four index patients with CMT (families M-P, Table3 and Fig.1). The variant in Family M, *NEFL* c.803T>G (p.L268R) affects the same amino acid as a previously reported mutation p.L268P (rs62636502) (Fabrizi et al. 2007). Family M presented with an autosomal dominant axonal CMT phenotype. The mutation was present in four affected individuals from three generations and not present in one unaffected at-risk individual. Bioinformatics predictions suggested p.L268R is likely to cause disease (Table3). The c.803T nucleotide is highly conserved and evolutionary constrained. These results support p.L268R to be pathogenic.

A second mutation, *NEFL* c.794A>G (p.Y265C) was identified in a family with axonal CMT (Family N). The proband had muscle weakness in the hands and distal legs, with sensory loss and neuropathic pain in the feet. While predominantly a peripheral neuropathy the patient showed clear upper motor neuron involvement with facial weakness. The disease showed autosomal dominant inheritance with a strong family history of neuromuscular disease. The c.794A>G sequence variant was confirmed in the proband and three additional affected individuals and was not present in one unaffected individual and one married-in spouse. While this nucleotide is conserved, bioinformatics analyses were contradictory making it difficult to determine the likely pathogenicity status of this variant (Table3).

The variant c.1007T>C (p.L336P) was identified in a patient with axonal CMT with asymmetrical gait (Family O). The inheritance pattern of the disease in this family is unclear and no other DNA samples were available for testing. Bioinformatics analysis suggested the variant is likely to cause disease (Table3). The p.L336P mutation is 4 and 3 residues respectively from two previously reported mutations (p.Q332P and p.L333P) causing CMT2 (Mersiyanova et al. 2000; Choi et al. 2004) which suggests this region may be important for NEFL function.

The novel sequence variant c.1319C>T (p.P440L) was identified in an autosomal dominant family with axonal CMT (Family P). Affected individuals had upper and lower limb muscle weakness and wasting, slight peroneal weakness (heel walk), brisk reflexes (upper and lower), and a possible extensor plantar response. Some individuals also had reduced sensory action potentials, whereas others had no sensory involvement. Segregation of p.P440L NEFL in the family is unclear. The variant was present in all tested affected individuals and absent in one unaffected (at-risk) individual. However, it was also present for an at-risk family member of unknown clinical phenotype. Some late onset cases have been described for the NEFL mutations (Fabrizi et al. 2007) and age-related penetrance may be involved in this family. Bioinformatics analysis support that p.440L is pathogenic as the residue is conserved across vertebrates and evolutionary constrained (Table3).

### Mutation in TRPV4

A novel *TRPV4* missense mutation c.549G>C (p.E183D) was identified in the index individual from Family Q (Table3 and Fig.1) diagnosed with an axonal motor and sensory neuropathy with retinal degeneration. The patient had wasting of the muscles of the feet and hands, sensory loss and reduced vibration sense. There was no scoliosis seen in this patient as reported for some *TRPV4* mutations (Auer-Grumbach et al. 2010; Deng et al. 2010). Family history, based on the patient's description, suggests an autosomal dominant inheritance, however segregation analysis was not possible, as no other family member samples were available for testing. Bioinformatics analysis of p.E183D indicated the glutamic acid residue is highly conserved across vertebrates, however the effect of this residue change on protein function was predicted unlikely to have a damaging effect by both SIFT and Polyphen2 (Table3). Autosomal dominant inheritance of a different mutation at the same amino acid position, p.E183K, has been reported to cause the unrelated disorder, spondylo-epimetaphyseal dysplasia Maroteaux-pseudo-Morquio type 2 (SEDM-PM2) (Nishimura et al. 2010). Our patient does not exhibit any overlapping clinical features with this disorder. Collectively, these data suggest that p.E183D is a rare polymorphism. More information, such as family segregation, will be required for a conclusive interpretation of pathogenicity of the c.549G>C p.E183D variant.

### Mutations in KIF1B

Two novel sequence variants were identified in *KIF1B;* NM_183416.3:c.2274G>T and NM_015074.3:c.4073T>C causing the missense mutations p.E758D and p.V1358A respectively. The index individual with the c.2274G>T variant is a male diagnosed with HMN with pyramidal signs (Family R, Table3 and Fig.1). He presented with pes cavus, hammertoes, muscle wasting of the feet and was unable to heel walk. The inheritance pattern in this family is unclear from the limited pedigree information. The proband has an affected brother but no clinical information or DNA sample was available for this individual. Segregation analysis was, therefore, not performed.

The family with the c.4073T>C sequence variant (Family S, Table3 and Fig.1) had autosomal dominant axonal CMT. Two affected sisters and their male cousin (on the mother's side) showed weakness of the feet muscles and moderate distal sensory loss. The mother of the two affected sisters was clinically examined and found to be asymptomatic suggesting reduced penetrance of the disease. All affected and nonpenetrant carrier individuals available for testing carried the p.V1358A *KIF1B* variant. Detailed clinical information and DNA samples for other family members were not available. Mutations in *KIF1B* that are associated with CMT have only been reported in one family (Zhao et al. 2001) so there is no precedent whether clinical heterogeneity will be observed with mutations in this gene. While the families reported here have two distinct IPN phenotypes, the family with c.4073T>C sequence variant has a similar phenotype to the original paper reporting *KIF1B* (*Saito* et al. 1997).

Assessing the likelihood of pathogenicity of the *KIF1B* variants using bioinformatics was, collectively, inconclusive. Although the base position c.2274T is evolutionarily constrained, its respective amino acid residue p.E758 is not strongly conserved in vertebrates. Both prediction programs SIFT and Polyphen2 suggested that the amino acid change p.E758D would be damaging. The second mutation, p.V1358, is highly conserved but not evolutionary constrained and though SIFT predicted p.V1358A would not be tolerated, Polyphen2 predicted this variant to be benign.

### Mutations in BICD2

Two novel missense mutations in *BICD2* were identified. Mutations in *BICD2* are generally associated with an autosomal dominant SMA characterized by an early onset, slowly progressive weakness and wasting of both proximal and distal muscles mainly affecting the legs (Neveling et al. 2013). A missense variant c.1079C>T (p.A360V) was identified in an individual with demyelinating CMT (Family T, Table3 and Fig.1). Autosomal dominant inheritance was inferred from patient information, with the proband's brother similarly affected and their mother has a sensory neuropathy without motor involvement. However, no additional DNA samples were available for testing which prevented segregation analysis.

The missense variant c.1993G>T (p.V665L) *BICD2* was identified in a family with an axonal hereditary motor neuropathy (Family U, Table3 and Fig.1). The proband (father of patient exomed) and his sister had pes cavus, hammertoes and pressure areas under the feet. At the time of clinical examination, there was no muscle wasting and nerve conduction velocities were within the normal range. The mutation is present in the two affected siblings and their affected father and not carried by their unaffected mother. An aunt on the father's side is reportedly also affected however a DNA sample was unavailable for testing.

Bioinformatics analysis suggested neither *BICD2* variant is likely to be pathogenic as the variants are not well conserved and both prediction programs suggested the variant changes are benign (Table3).

### A previously reported SETX mutation is unlikely to be pathogenic

The sequence variant *SETX*:c.7640T>C (p.I2547T) variant (Table S1) was previously reported as a putative cause of a sporadic ataxia-tremor and motor neuron disease phenotype in a single individual (Hirano et al. 2011). We identified the same sequence variant in a family with a CMT2 phenotype (Family V, Fig.1). The variant did not segregate with disease in the CMT2 family (absent in five out of seven affected individuals) and was found to be originally inherited from a married-in unaffected individual. Therefore, c.7640T>C is likely to be a rare polymorphism and not the cause of the disease in our family. These data also suggest it is unlikely the pathogenic lesion in the previously reported single ataxia/neuropathy case.

### Rare polymorphisms

We identified an additional 20 sequence variants that were excluded as nonpathogenic variants and are likely to be rare polymorphisms or private variants (Table S1). The following criteria were used to assign a variant as a polymorphism (Macarthur et al. 2014): affected family member(s) not carrying the sequence variant, evidence the variant is a reported polymorphism, the variant is inherited from a married-in individual, or assuming high penetrance for CMT, two or more unaffected at-risk individuals carry the variant.

## Discussion

We have identified mutations in nine families that have been previously reported to be pathogenic in known IPN genes. Following current guidelines for assigning pathogenicity (Macarthur et al. 2014), with the segregation results and evidence from the literature, we consider the previously reported mutations as causative of disease in these families. A further 12 novel sequence variants (Table3) were identified that segregated in families and were absent from 1000 neurologically normal control chromosomes. Conservation and bioinformatics analysis showed additional supportive evidence implicating six of these variants as causing disease in these families. The remaining novel sequence variants are unsupported or inconclusive by these analyses and are unlikely causing the disease in these families. However, as the genetic evaluation of these novel variants has reached the limits of the small families (there is no more power in the pedigree) these results remain inconclusive and we are at an impasse with the variants identified in these families.

In our study, mutations in known genes accounted for 8% (9 of 110) of the cohort, with an additional 11% (12 of 110) with novel sequence variants that are yet to be proven pathogenic. Prior genetic testing has identified most patients that have mutations in common genes. The overall contribution of the known disease genes in CMT previously published is 67%, with over 90% of this genetic diagnosis described by four common genes (Saporta et al. 2011). The remaining CMT disease genes are rare and account for only a few cases. In a study considering the HMN subtype of IPN, there was no gene accounting for a majority of cases, with known genes accounting for only 15% of disease (Dierick et al. 2008). Therefore, the yield of successful genetic diagnosis in this study is similar to previous studies. The remaining cases with unknown genetic diagnosis (81%) will form the basis of future studies to identify new IPN genes and no variants of unknown significance were identified in these patients.

Six of the mutations identified in this study were in genes that are routinely screened as part of normal diagnostic procedures (*MFN2*; 2 families and *GJB1*; 4 families). On review, both *MFN2* mutations were identified in families last clinically examined prior to *MFN2* testing becoming available, and this study was the first genetic review of each family. Of the families with *GJB1* mutations, none had male-to-male inheritance. In three families, X-linked inheritance was not previously recognized due to no affected males with sons and a limited number of males and females (or no males) for phenotype variability to effectively assign this mode of inheritance. The remaining family with *GJB1* was suspected of being X linked and previously tested for *GJB1* (year 1997), with a negative result which is likely due to reduced sensitivity of mutation scanning methods used at that time. The implementation of WES in a diagnostic setting is unlikely to increase the overall genetic diagnostic success rate (based on standard MLPA/Sanger sequencing methodologies), but in future may lead to reduced time and costs for samples prescreened for the CMT1A duplication (Choi et al. 2012). WES will likely increase the yield of private novel sequence variants in the rare CMT genes that cannot be conclusively assigned pathogenicity (in small pedigrees). This is of increasing importance, as genetic diagnosis now carries a greater ethical burden as highlighted in a recent review (Rossor et al. 2013).

In this study, 12 novel sequence variants were identified in families as a possible cause of disease. While all were absent from 1000 neurological normal control chromosomes, supportive evidence from bioinformatics and conservation was obtained for only six sequence variants. In these cases the sequence variant is likely to be the correct disease variant. For the remaining novel variants without supportive bioinformatics evidence it is unclear if they represent rare private variants unrelated to the disease or if the bioinformatics results are in error. To further highlight the need for caution when interpreting novel sequence variants in known disease genes, this study identified the sequence variant *SETX*:c.7640T>C (p.I2547T) which was previously reported as a putative cause of a sporadic ataxia-tremor and motor neuron disease phenotype in a single individual (Hirano et al. 2011). With the additional evidence presented here this variant is unlikely to be pathogenic. The list of rare polymorphisms identified in this study (Table S1) may help other researchers who are unable to interpret the pathogenic impact of candidate variants in small families with IPN.

We used the mean depth of coverage as a measure of the sensitivity of WES to identify mutations. We estimated the sensitivity to detect mutations in IPN genes in this cohort is in the range of 90–98% (Choi et al. 2009). The sample with the lowest quality sequencing (average read depth of 27.5X), was considered less than ideal, nevertheless, a *MFN2* mutation was successfully identified in this sample (Family B). Additionally, we correctly identified the mutation for two positive control samples as well as *AARS* SNPs (McLaughlin et al. 2012) in three patients (Table S1) that had previously been detected through a mutation screen of all aminoacyl tRNA-synthetase genes (McLaughlin et al. 2012). Not all exons targeted by WES are covered sufficiently for gene screening (Montenegro et al. 2011). As we did not resequence gaps using an alternative method, it is possible that a small number of additional mutations may be identified within these regions. In addition, it is possible that copy number and structural variations in known genes may provide some additional diagnosis, but this is likely to be rare (Huang et al. 2010). Examination of mitochondrial genes such as *MT-ATP6* (Pitceathly et al. 2012) can also be carried out in families with possible maternal inheritance patterns of disease.

In conclusion, we have successfully applied WES to our cohort of IPN cases and identified pathogenic sequence variants in 8% of cases and novel sequence variants that may cause disease in an additional 11% of our cohort. This study has highlighted the advantage of using WES for genetic diagnosis in highly heterogeneous diseases such as the IPNs and has been especially powerful when inheritance is unclear. However, first tier testing for common genes in clinically well-defined cases remains important and will account for most positive results. Research into the genetic cause of the remaining cases will identify new disease genes furthering our understanding of the biology underlying IPNs.

## Supporting Information

**Figure S1.** (A–D) Mean percentage of the CDS covered by WES in 112 samples at defined read-depths of 1X, 10X, and 20X. Error bars show 95% confidence interval.

**Table S1.** Rare polymorphisms identified in known IPN genes.
