# Supplementary material for: Improved inherited peripheral neuropathy genetic diagnosis by whole-exome sequencing
Source: Mol Genet Genomic Med. 2015 Jan 14;3(2):143–54. doi: 10.1002/mgg3.126 (PMC4367087; doi:10.1002/mgg3.126)
Supplement: Supplementary file 2 [file mgg30003-0143-sd2.pdf]

C

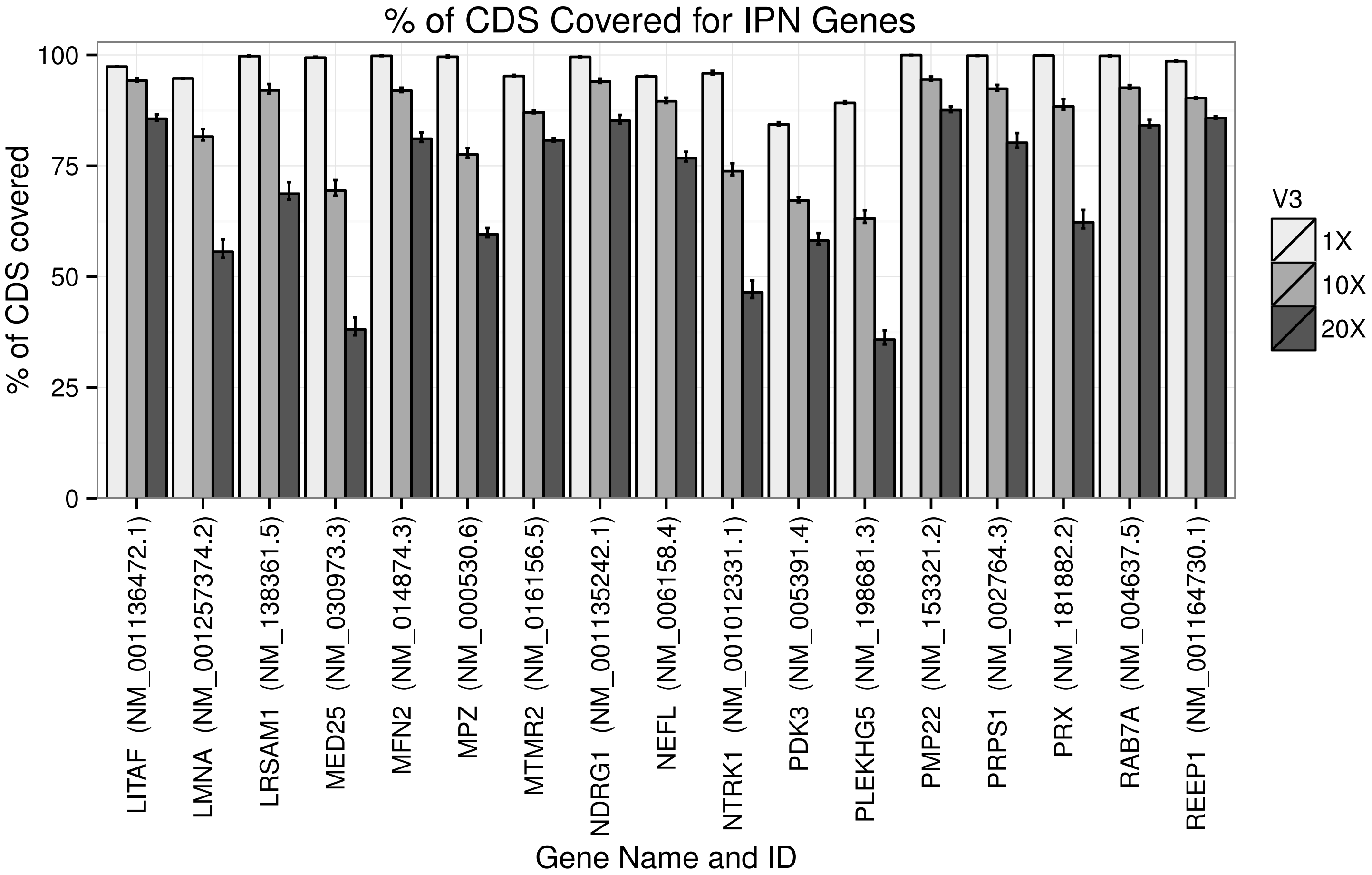

D

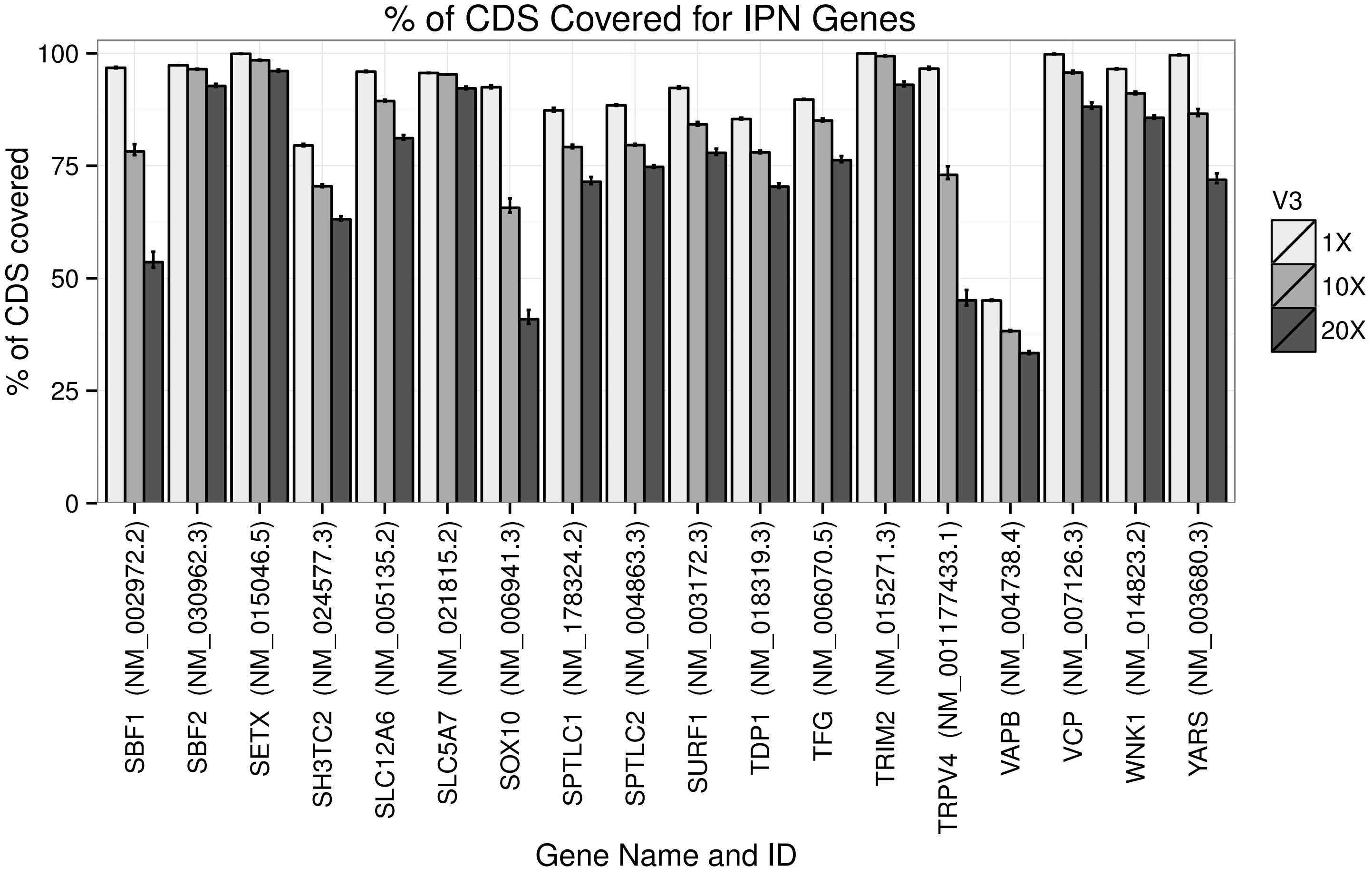

Supplementary Figure S1 C - D: Mean percentage of the CDS covered by WES in 112 samples at defined read-depths of 1X, 10X and 20X. Error bars show 95% confidence interval.
