# Supplementary material for: Improved inherited peripheral neuropathy genetic diagnosis by whole-exome sequencing
Source: Mol Genet Genomic Med. 2015 Jan 14;3(2):143–54. doi: 10.1002/mgg3.126 (PMC4367087; doi:10.1002/mgg3.126)
Supplement: Supplementary file 3 [file mgg30003-0143-sd3.docx]

**Supplementary Tables**

Table S1: Rare polymorphisms identified in known IPN genes.

| **Disease Classification** | **Gene** | **cDNA change** | **Amino acid change** | **dbSNP accession** | **Evidence** | **Reference** |
| --- | --- | --- | --- | --- | --- | --- |
| CMT2 | *SETX* | c.7640T>C | p.I2547T |  | **A** | ([Hirano et al., 2011](#_ENREF_21)) |
| CMT2 | *NEFL* | c.289C>T | p.L97F |  | A |  |
| CMT2 | *KIF1B* | c.3269T>C | p.I1090T |  | M |  |
| CMT1 | *FIG4*** | c.122T>C | p.I41T | rs121908287 | P | ([Chow et al., 2007](#_ENREF_10)) |
| CMT2 | *GDAP1* | c.248G>C | p.G83A |  | M |  |
| CMT + Pyramidal signs | *AARS* | c.1685C>T | p.T562I | rs148355156 | P A | ([McLaughlin et al., 2012](#_ENREF_32)) |
| HMN | *AARS* | c.2185C>T | p.R729W* | rs138081804 | P | ([McLaughlin et al., 2012](#_ENREF_32)) |
| CMT2 | *AARS* | c.2791G>A | p.G931S* |  | P | ([McLaughlin et al., 2012](#_ENREF_32)) |
| CMT2 | *AARS* | c.2791G>A | p.G931S* |  | P | ([McLaughlin et al., 2012](#_ENREF_32)) |
| CMT1 | *LRSAM1* | c.1046A>G | p.Q349R |  | A |  |
| Spastic Paraplegia + CMT2 | *BICD2* | c.2296C>T | p.R766W |  | M |  |
| HMNP | *BSCL2* | c.833C>T | p.P278L | rs17850877 | A |  |
| HMNP | *SETX* | c.1869A>C | p.E623D | rs139200312 | A |  |
| HMNP | *SETX* | c.3968G>A | p.R1323Q |  | A |  |
| HMN | *SETX* | c.7432A>C | p.T2478P | rs142303658 | m P |  |
| CMT2 | *SETX* | c.7640T>C | p.I2547T |  | A | ([Hirano et al., 2011](#_ENREF_21)) |
| HMN | *DCTN1* | c.3620C>T | p.T1207I | rs72466496 | P | ([Munch et al., 2004](#_ENREF_35)) |
| Intermediate CMT | *TRPV4* | c.1570T>G | p.F524V |  | A |  |
| CMT2 + Pyramidal signs with cough | *YARS* | c.241G>C | p.D81H |  | A |  |
| CMT2 | *DCTN1* | c.3620C>T | p.T1207I | rs72466496 | P | ([Munch et al., 2004](#_ENREF_35)) |

Amino acid position is relative to the following GenBank Accession numbers. *SETX*: NM_015046.5, *NEFL*: NM_006158.4, *KIF1B*: NM_015074.3, *FIG4*: NM_014845.5, *GDAP1*: NM_018972.2, *AARS*: NM_001605.2, *LRSAM1*: NM_138361.5, *BICD2*: NM_015250.3, *BSCL2*; NM_001130702.2, *DCTN1*; NM_001190836.1, *TRPV4*: NM_021625.4, *YARS*: NM_003680.3 . Evidence, A: Affected family member(s) does not carry the mutation. M: Two or more unaffected at risk individuals carry the mutation. m: one unaffected at risk individual carries the mutation. P: Published evidence of polymorphism. *Variant detected in that particular patient in a previous study ([McLaughlin et al., 2012](#_ENREF_32)). **Patient is a heterozygous carrier from a family with autosomal dominant mode of inheritance.
